# Stimulus‐dependent sex differences in dyspnoea: Mechanical loading vs. hypercapnia

**DOI:** 10.1113/EP094211

**Published:** 2026-09-27

**Authors:** Jack R. Dunsford, Olga Radivojevic, Olivia N. Ferguson, Alanna S. Hind, Michele R. Schaeffer, Owen D. Harris, Satvir S. Dhillon, Kathryn M. Milne, Dennis Jensen, Paolo B. Dominelli, Yannick Molgat‐Seon, Jordan A. Guenette

**Affiliations:** ^1^ Centre for Heart Lung Innovation, Providence Research The University of British Columbia (UBC) and St. Paul's Hospital Vancouver British Columbia Canada; ^2^ Department of Physical Therapy UBC Vancouver British Columbia Canada; ^3^ Division of Respiratory Medicine UBC Vancouver British Columbia Canada; ^4^ Department of Kinesiology and Physical Education McGill University Montreal Quebec Canada; ^5^ Faculty of Kinesiology University of Calgary Calgary Alberta Canada; ^6^ Department of Kinesiology and Applied Health University of Winnipeg Winnipeg Manitoba Canada

**Keywords:** air hunger, dyspnoea, inspiratory muscles, sex differences

## Abstract

Dyspnoea is a multidimensional sensation of breathing discomfort that varies in sensory and affective experience. During exercise, healthy adults commonly report dyspnoea sensations of ‘air hunger’ and ‘work/effort’, with females more frequently reporting air hunger at maximal exertion. Interpreting these sex differences and their underlying mechanisms is challenging due to the complex physiological responses associated with exercise. Therefore, this study used laboratory models to target respiratory stimuli and evaluate sex‐differences in perceptual and physiological responses to dyspnoea. In a randomized crossover design, 34 healthy participants (*n* = 17M; *n* = 17F) completed two breathing challenges. Work/effort was targeted using incremental inspiratory pressure threshold loading (IPTL). Air hunger was targeted by constraining ventilation while incrementally increasing the partial pressure of end‐tidal carbon dioxide ($P_{\mathrm{ETC}O_{2}}$). Ratings of dyspnea intensity, unpleasantness, work/effort and air hunger were assessed at the end of each increment. Electromyography of the crural diaphragm (EMG_di_), sternocleidomastoid (EMG_scm_) and scalene (EMG_sca_) were measured throughout each trial. Mixed‐effects models tested sex‐by‐stimulus interactions. During IPTL, males exhibited a significantly steeper increase in dyspnoea intensity and work/effort ratings than females. These perceptual differences occurred without a concomitant sex difference in EMG_di,_ while EMG_scm_ and EMG_sca_ were lower in males compared to females. During hypercapnia, there were no sex‐by‐stimulus interactions for perceptual or physiological outcomes, despite significantly greater EMG_scm_ in females than males. These findings highlight that sex differences in dyspnoea are stimulus‐dependent, occurring during mechanical loading but not chemostimulation, and may partly reflect differences in perceptual anchoring to experience and expectation.

## Abstract (highlights)

- **What is the central question of this study?**
  Females more often report the dyspnoeic sensation of air hunger than males: what are the mechanisms of this sex difference?
- **What is the main finding and its importance?**
  During inspiratory pressure threshold loading, males showed a steeper increase in ratings of dyspnoea intensity and work/effort but similar dyspnoea unpleasantness and air hunger, despite similar diaphragm activation and lower sternocleidomastoid and scalene activity compared to females. During hypercapnia, no sex differences were observed for any perceptual outcomes. Sex differences in dyspnoea are stimulus‐dependent, present during mechanical loading but absent during hypercapnia, and may reflect perceptual anchoring to prior experience and expectation.

## 1 INTRODUCTION

Dyspnoea is a multidimensional sensation of breathing discomfort that varies in quality, intensity, unpleasantness and emotional response (Banzett et al., 2015; Parshall et al., 2012). Dyspnoea, defined as a score of 2+ on the modified Medical Research Council breathlessness scale (Bestall et al., 1999), is reported in 3.6–11% of adults in the general population (Bowden et al., 2011; Kochovska et al., 2024) with prevalence increasing with age to 35% in individuals over 70 years (Ho et al., 2001). Dyspnoea is an important health outcome measure and an independent predictor of both morbidity and mortality in the general population (Abidov et al., 2005); it is associated with worse psychological health and greater functional limitations and is a major contributor to poor adherence to exercise programmes in sedentary adults and patients with respiratory disease (Hayton et al., 2013; Ho et al., 2001).

Different dyspnoea sensations arise from distinct stimuli and afferent inputs, producing qualitatively different perceptual experiences (Lansing et al., 2009; O'Donnell et al., 2013; Parshall et al., 2012). Dyspnoea perception can be broken down into the sensory dimension, which encompasses the perception of quality and intensity, and the affective dimension, which includes the level of unpleasantness or discomfort and the emotional response (Banzett et al., 2015; Lansing et al., 2009; Parshall et al., 2012). The two most common sensory qualities of dyspnoea are work/effort and air hunger, each resulting from different neurophysiological mechanisms (Banzett et al., 2015; Lansing et al., 2009). Work/effort is associated with an awareness of increased central respiratory motor command (el‐Manshawi et al., 1986; Gandevia & Macefield, 1989), whereas air hunger is primarily associated with an inadequate ventilatory response to increased neural respiratory drive (NRD), also known as neuromechanical dissociation (NMD) (Faisal et al., 2016; Laveneziana et al., 2011; O'Donnell, 2005). These qualities elicit different affective responses. For example, sensations of air hunger are associated with greater unpleasantness and a stronger emotional response than the work/effort quality (Banzett et al., 2008).

During exercise, females report higher ratings of overall dyspnoea intensity and unpleasantness at any absolute, but not relative, minute ventilation (${\dot{V}}_{E}$) or work rate compared to males (Cory et al., 2015; Ofir et al., 2008; Schaeffer et al., 2014). Indeed, when ${\dot{V}}_{E}$ or work rate are measured relative to individual maximal capacity, these sex differences disappear (Mitchell et al., 2026; Molgat‐Seon et al., 2018). However, females more frequently report qualitative sensations of dyspnoea related to unsatisfied inspiration at maximal exercise (Cory et al., 2015), which is linked to increased unpleasantness and a stronger emotional response compared to sensations of work and effort (Banzett et al., 2008). These sex differences in exertional dyspnoea may reflect, at least in part, sex‐related differences in respiratory system morphology. For example, proportionally smaller lungs and airways in females compared to males have been proposed to impose a relatively greater mechanical constraint in females, which may increase the likelihood of an inadequate ventilatory response (Dominelli & Molgat‐Seon, 2022; Schaeffer et al., 2014). However, Molgat‐Seon *et al.* (2019) experimentally reduced mechanical constraint during exercise through an inspired helium–oxygen mixture and showed no change in dyspnoea ratings compared to ambient air breathing in males and females. Indeed, the precise mechanisms underlying the sex difference in air hunger perception at peak exercise remain unclear.

During exercise, respiratory and locomotor muscle afferent signals are tightly coupled, making it difficult to experimentally dissociate their respective contributions to dyspnoea. Laboratory‐induced dyspnoea using non‐exercise respiratory stimuli enables precise manipulation of breathing parameters to elicit distinct dyspnoea qualities while minimizing confounding input from locomotor muscle afferents. Sensations of work/effort can be induced in a laboratory setting by adding inspiratory threshold loads (Rolland‐Debord et al., 2026; Simon et al., 1989). Sensations of air hunger can be induced through increasing the chemical drive to breathe by increasing the inspired fraction of carbon dioxide ($F_{\mathrm{IC}O_{2}}$) while limiting tidal volume (*V*_t_) and breathing frequency (*f*_b_) through voluntary hypopnea (Banzett et al., 2008; O'Donnell et al., 2013). These laboratory models allow for more selectively weighted dyspnoeic stimuli targeting the specific dyspnoea qualities.

We aimed to evaluate sex differences in the perception of dyspnoea using laboratory models known to predominantly target the mechanisms of the work/effort and air hunger qualities of dyspnoea and to characterize the underlying physiological mechanisms in males and females. We hypothesized that when targeting sensations of work/effort, males and females would not differ in their perceptual response; however, when targeting sensations of air hunger, ratings of dyspnoea would be higher in females compared to males with a concomitant increase in NMD in females.

## 2 METHODS

### 2.1 Ethics approval

All participants provided written informed consent. The study received institutional ethical approval from The University of British Columbia – Providence Health Care Research Ethics Board (H24‐01587), and adheres to the *Declaration of Helsinki*, except for registration in a database.

### 2.2 Participants

Thirty‐four healthy adults (age range: 19–35 years) participated in this study. Participants had normal spirometry (i.e., forced expired volume in 1 s (FEV_1_)/forced vital capacity (FVC) ratio ≥0.70) and a body mass index ≥18 and ≤30 kg m^−2^ and reported having no history of cardiopulmonary disease, smoking or contraindications to an oesophageal catheter.

### 2.3 Experimental overview

The visit began with participants completing the Hospital Anxiety and Depression Scale questionnaire (Zigmond & Snaith, 1983), collecting height and body mass, pulmonary function testing performed according to standard recommendations (Graham et al., 2019), and familiarization with symptom scales. After placement of an oesophageal catheter (see ‘Physiological measurements’), participants completed maximal inspiratory (MIP) and expiratory (MEP) pressure manoeuvres (Laveneziana et al., 2019), as well as inspiratory capacity manoeuvres (Guenette et al., 2013). Participants then completed two incremental breathing challenges: inspiratory pressure threshold loading (IPTL) and hypercapnic expiratory resistive breathing (HERB). The increments in dyspnoea stimulus for both trials were made relative to the participant to promote similar within‐trial duration between the sexes. The trials were separated by a minimum of 20 min of rest, with order of the trials randomized and counterbalanced to ensure an equal distribution of males and females for each trial order. The multiple dimensions of dyspnoea were assessed throughout each trial. Participants were blinded to the sex difference focus of the study to limit expectation bias in reporting perceptions of dyspnoea. Instead, the purpose of the study was explained as ‘characterizing the multidimensional perceptions of breathlessness in response to different breathing challenges’. Following data collection, participants were debriefed by providing further explanation of the purpose of the study including the analysis of sex differences.

### 2.4 Inspiratory pressure threshold loading

**FIGURE 1:**
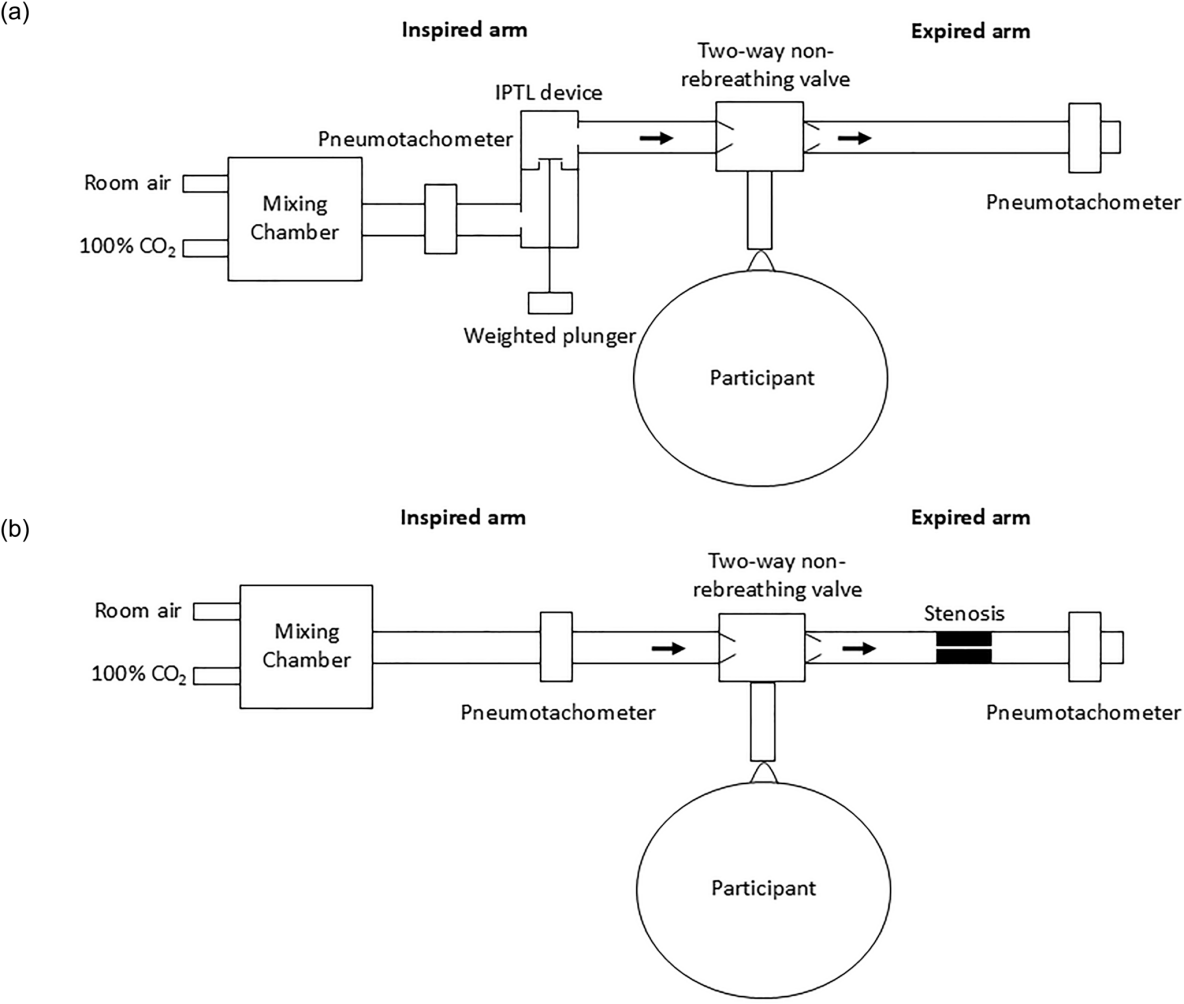
Apparatus diagrams for IPTL (a) and HERB (b). IPTL, inspiratory pressure threshold loading; HERB, hypercapnic expiratory resistive breathing.

The purpose of this trial was to incrementally increase the work of breathing while maintaining resting partial pressure of end tidal carbon dioxide ($P_{\mathrm{ETC}O_{2}}$). To achieve this, participants breathed through a two‐way non‐rebreathing valve (Model 2700, Hans Rudolph, Shawnee, KS, USA) with a custom‐built IPTL device attached to the inspiratory arm (see Figure 1a), based on the design by Nickerson & Keens (1982). While breathing on the device, participants were required to generate sufficient inspiratory pressure to lift a weighted plunger and allow air to enter the circuit. The trial began with a baseline of 10 min of resting breathing with no resistance. Each subsequent stage lasted 90 s, with the first stage consisting only of the resistance imposed by the weight of the plunger. A small basket (31 g) was added to the plunger at the beginning of the second stage. In the third stage, a specific weight was added to increase the threshold pressure to 10% of the participant's MIP. Weight was then added every 90 s to increase threshold pressure by ∼5% of MIP until the participant was unable to sufficiently lift the plunger for three consecutive breaths or dyspnoea became intolerable. Participants breathed at a predetermined duty cycle of 1 s:1 s (inspiration:expiration), paced by a metronome. $P_{\mathrm{ETC}O_{2}}$ was monitored on a breath‐by‐breath basis and titrated by adding 100% CO_2_ into the inspired portion of the breathing circuit as needed to maintain normocapnia.

### 2.5 Hypercapnic expiratory resistive breathing

The purpose of this trial was to maintain ${\dot{V}}_{E}$ near resting levels while incrementally increasing chemostimulation via controlled increases in $P_{\mathrm{ETC}O_{2}}$. To maintain ${\dot{V}}_{E}$, we adapted the expiratory resistive breathing method developed by Urban *et al.* (2021). Briefly, we combined resistance to expiration (via a stenosis placed in the expiratory arm of a two‐way non‐rebreathing valve; see Figure 1b) and a metronome paced duty‐cycle of 1 s:3 s (inspiration:expiration). We then instructed participants to maintain expiratory mouth pressure (*P*_mo_) at ∼5% maximal expiratory pressure for each expired breath throughout the trial. We provided real time verbal instruction to increase, decrease or maintain expiratory effort on a breath‐by‐breath basis to help participants achieve and maintain the target *P*_mo_. Together, the fixed expired duration and duty cycle at a fixed expired *P*_mo_ resulted in a consistent ${\dot{V}}_{E}$.

Before the trial began, resting ${\dot{V}}_{E}$ and $P_{\mathrm{ETC}O_{2}}$ were determined during 10 min of relaxed unconstrained breathing while viewing a nature documentary to minimize conscious breathing alterations. We then selected an expiratory resistance (a stenosis with opening of 2.8–3.6 mm) that, when combined with the 3‐s expiratory breathing time and target *P*_mo_, produced an expired volume sufficient to maintain ${\dot{V}}_{E}$ at 20% above rest. The selected level of resistance was confirmed during 2 min of normocapnic, metronome‐paced breathing with the expiratory resistance in place.

The trial began with 3 min of breathing following the metronome paced 1 s:3 s duty cycle. $P_{\mathrm{ETC}O_{2}}$ was then increased by ∼10% of rest by titrating 100% CO_2_ into the inspired arm of the breathing circuit. Each increment was held for 3 min once a steady state in $P_{\mathrm{ETC}O_{2}}$ was established to allow for central chemoreceptor equilibration (Banzett, 1996). Trials continued until either (1) breathing discomfort became intolerable, (2) the participant could not maintain the appropriate *V*_t_ for three consecutive breaths, or (3) a $P_{\mathrm{ETC}O_{2}}$ of 60 mmHg was reached.

### 2.6 Dyspnoea measurement

In the last 30 s of each increment of each trial, participants were asked to rate their dyspnoea intensity, dyspnoea unpleasantness, the intensity of the sensation of work/effort and the intensity of the sensation of air hunger on the 0–10 category‐ratio (CR10) Borg scale (Borg, 1982), whereby a rating of ‘0’ represented no sensation of relevant quality (i.e., intensity, unpleasantness, work/effort, air hunger) and ‘10’ represented the greatest sensation of the relevant quality they have ever experienced or could ever imagine experiencing. Standardized scripts were used to differentiate intensity from unpleasantness (Price et al., 1983) and work/effort from air hunger (Lansing et al., 2000). Immediately following each trial, participants completed the Multidimensional Dyspnea Profile (MDP) (Banzett et al., 2015) with an explicit focus on how these sensations were experienced at the very end of the trial immediately before removing the mouthpiece.

### 2.7 Flow, volume and $P_{\mathrm{ETC}O_{2}}$

Inspired and expired airflow were measured continuously using independent heated pneumotachometers (Models 3813 and 4813, Hans Rudolph). The integral of the airflow signal provided a measure of inspired and expired volume. $P_{\mathrm{ETC}O_{2}}$ was measured via a sampling port just distal to the expired valve and analysed by an infrared CO_2_ analyser (Model 12630, VacuMed, Ventura, CA, USA).

### 2.8 Electromyography

Electromyography of the crural diaphragm (EMG_di_) was measured via a multipair oesophageal electrode catheter connected to a bio‐amplifier (bio‐amplifier model RA‐8; Yingui Medical Technology Co. Ltd, Guangzhou, China) as previously described by Luo *et al.* (2008). To measure activity of the extradiaphragmatic inspiratory muscles, wired surface electrodes were placed on the midway point between the mastoid process and the medial clavicle in the centre of the muscle belly (Shadgan et al., 2011), and in line with the cricoid cartilage in the posterior triangle of the neck (Ramsook et al., 2017), to measure EMG of the sternocleidomastoid (EMG_scm_) and scalene (EMG_sca_), respectively. All raw EMG measures were converted to root mean square (RMS) using a time constant of 100 ms and a moving window. EMG was measured as the maximum RMS value of each inspired breath by selecting values which fell between QRS complexes on the EMG_di_ signal to avoid artifacts from the electrical activity of the heart. EMG is presented as the percentage of the largest RMS value achieved during inspiratory capacity manoeuvres.

### 2.9 Neural respiratory drive and neuromechanical dissociation

EMG_di_%max was used as a surrogate to estimate NRD as described previously (Lourenço et al., 1966; Ramsook et al., 2017). Given that extradiaphragmatic inspiratory muscle activation increases with NRD (Niro et al., 2021), EMG_scm_ and EMG_sca_ were evaluated as additional measures of inspiratory muscle activation alongside EMG_di_. NMD was estimated as a ratio of EMG_di_%max to *V*_t_ as a percentage of FVC (i.e., EMG_di_%max:*V*_t_%FVC) (Lewthwaite & Jensen, 2021).

### 2.10 Mouth pressure

During both trials, *P*_mo_ was measured using a calibrated pressure transducer (model DP15–34, Validyne Engineering, Northridge, CA, USA). *P*_mo_ is reported as a percentage of maximal *P*_mo_ achieved during MIP manoeuvres (*P*_mo_%MIP), wherein participants inhaled maximally from functional residual capacity against a semi‐occluded mouthpiece. *P*_mo_%MIP was used as a measure of relative inspiratory effort during the IPTL trial.

### 2.11 Data analysis

All physiological data were sampled at 2000 Hz and recorded using a PowerLab 16 channel analog‐to‐digital converter running LabChart Pro v8.1.17 software (ADInstruments, Colorado Springs, CO, USA). Manually selected 30 s segments of flow, volume, mouth pressure and EMG data for each stage of each trial were analysed using a custom Python script (Python version 3.12, 2023; Python Software Foundation, Wilmington, DE, USA). For all trials, the script removed electrocardiogram artifacts and calculated RMS from the EMG signal for each inspiratory breath. Additionally, for the HERB trials, the script calculated inspiratory capacity by correcting for volume signal drift to measure changes in operating lung volumes during the trial.

### 2.12 Statistical analysis

A minimum sample size of 32 (16 male, 16 female) was required for adequate power (80%) to detect a significant difference in dyspnoea intensity and unpleasantness (CR10 scale) between the two groups based on a clinically significant difference of 1 CR10 unit (Ries, 2005), a standard deviation of ±1 for CR10 rating changes (Ofir et al., 2008), 𝛼 = 0.05, and a two‐tailed test of significance. Comparisons between males and females for baseline characteristics were made using an unpaired Student's *t*‐test.

Generalized mixed effects modelling was used to estimate the effect of sex on the perceptual and physiological responses across each condition using the *GlmmTMB* (Brooks et al., 2017) and *lmer* (Bates et al., 2015) packages in R. Responses to each of the four dyspnoea questions (intensity, unpleasantness, work/effort and air hunger) were modelled using a β‐regression mixed effects model with logit link to account for the bounded nature of CR10 scales. To accommodate the β‐distribution's support of values between 0 and 1 (exclusive), the data were transformed to a 0–1 scale with scores of 0 and 10 adjusted to 0.0001 and 0.999, respectively. To estimate the physiological response to each dyspnoea stimulus, linear or quadratic mixed effects models were used. The model type (i.e., linear or quadratic) was selected based on physiological expectation, visual examination and comparison of Akaike's information criterion and the Bayesian information criterion (Van der Elst et al., 2016). For all perceptual and physiological models, the fixed effects included the interaction between sex and relative dyspnoea stimulus level, while participant ID was included as a random effect for both slope and intercept. Pairwise estimated marginal means were contrasted between sexes in increments of 5% from 0% up to and including the group mean peak *P*_mo_%MIP for IPTL and 10% increments from 0% up to and including the group mean peak %Δ$P_{\mathrm{ETC}O_{2}}$ for HERB using the *emmeans* package in R (Lenth et al., 2025). For all models and estimated marginal means, females were used as the reference group, and therefore all coefficients and contrasts represent the difference in males from females.

The A1, S1 and A2 components of the MDP were scored and independently tested for sex differences for each trial via a Mann–Whitney *U*‐test. The SQ component of the MDP was compared between sexes using Fisher's exact test.

## 3 RESULTS

### 3.1 Participant characteristics

A total of 34 participants (*n* = 17 males; *n* = 17 females) completed both experimental trials. Two participants (1 male, 1 female) could not tolerate the HERB trial and were excluded due to insufficient data; therefore, *n* = 32 (*n* = 16 males; *n* = 16 females) were included in all HERB analysis. All 34 participants were included in the IPTL analysis.

Descriptive characteristics of all males and females are shown in Table 1. Although males and females were similar in age (*P* = 0.89), males were significantly taller (*P *< 0.001), heavier (*P *< 0.001) and had a greater BMI (*P* = 0.005) than females. Males had greater absolute FVC (*P *< 0.001), FEV_1_ (*P *< 0.001), MIP (*P* = 0.004) and maximal expiratory pressure (*P *< 0.001) compared to females, but were well matched for lung function (Quanjer et al., 2012) and respiratory muscle strength when expressed as a percentage of race‐neutral predicted normal values (Lista‐Paz et al., 2023) (all *P *> 0.05). Males and females did not differ in their trait anxiety and depression scores (both *P *> 0.05).

**TABLE 1:** Participant characteristics.

Characteristic	Male (*n* = 17)	Female (*n* = 17)	*P*
Age (years)	24 ± 4	24 ± 3	0.89
Height (cm)	177 ± 8	166 ± 6	<0.001^*^
Body mass (kg)	76 ± 10	60 ± 7	<0.001^*^
BMI (kg/m^2^)	24 ± 3	22 ± 2	0.005^*^
FVC (L)	5.21 ± 0.81	3.95 ± 0.6	<0.001^*^
FVC (% predicted)	103 ± 11	106 ± 11	0.37
FEV_1_ (L)	4.34 ± 0.68	3.34 ± 0.46	<0.001^*^
FEV_1_ (% predicted)	101 ± 11	103 ± 10	0.50
FEV_1_/FVC (%)	83 ± 4	85 ± 5	0.42
FEV_1_/FVC (% predicted)	98 ± 5	97 ± 6	0.53
MIP (cmH_2_O)	−128 ± 25	−102 ± 24	0.004^*^
MIP (% predicted)	96 ± 19	97 ± 23	0.88
MEP (cmH_2_O)	164 ± 37	120 ± 26	<0.001^*^
MEP (% predicted)	88 ± 21	79 ± 17	0.10
Anxiety (0–21)	6.47 ± 3.84	8.76 ± 3.42	0.08
Depression (0–21)	2.29 ± 1.83	2.53 ± 1.87	0.71

All values are presented as means ± SD. *Significant difference between sexes. Abbreviations: BMI, body mass index; FVC, forced vital capacity; FEV_1_, forced expired volume in 1 s; MEP, maximal expiratory pressure; MIP, maximal inspiratory pressure.

### 3.2 Perceptual responses to IPTL

#### 3.2.1 CR10 ratings of dyspnoea

**FIGURE 2:**
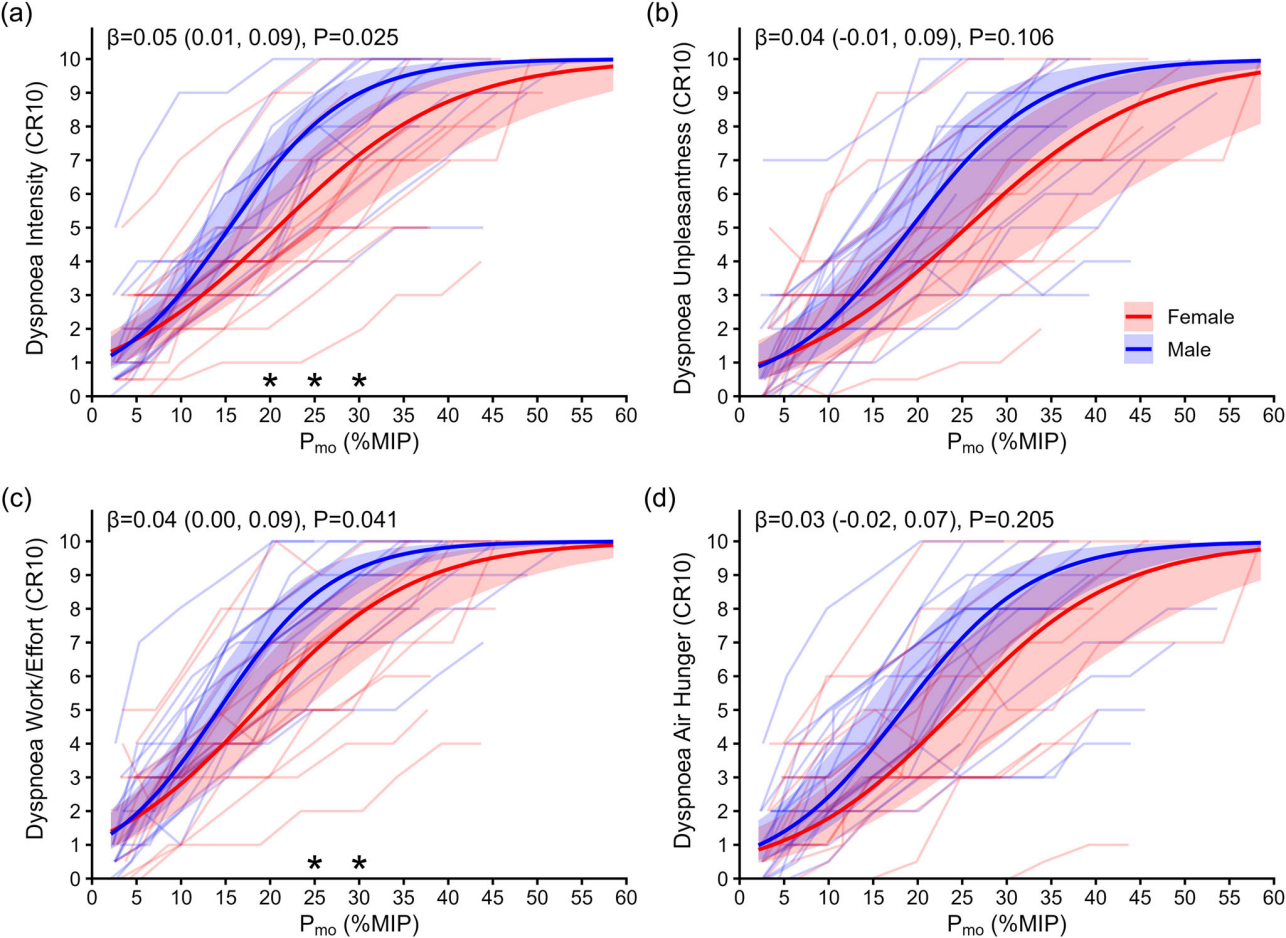
Dyspnoea responses during IPTL in males and females. (a) Rating of dyspnoea intensity. (b) Rating of dyspnoea unpleasantness. (c) Intensity of the descriptor work/effort. (d) Intensity of the descriptor air hunger. Bold lines and shaded areas (95% CIs) represent model estimates and thin lines represent individual participant responses at each increment of *P*_mo_%MIP. β, change in log‐odds for males compared to females per unit change in *P*_mo_%MIP. *P < 0.05 statistically significant between sexes estimated marginal mean contrast. CR10, 0–10 category ratio scale; IPTL, inspiratory pressure threshold loading; MIP, maximal inspiratory pressure; *P*_mo_, mouth pressure.

There was a significant interaction between sex and *P*_mo_%MIP for dyspnoea intensity (*P* = 0.025) (Figure 2a) and for work/effort (*P* = 0.041) (Figure 2c) whereby males exhibited a steeper rise in intensity and work/effort ratings per unit increase in *P*_mo_%MIP compared to females. Pairwise estimated marginal mean contrasts showed significantly higher dyspnoea intensity ratings in males at 20% (1.85 CR10 [CI: 0.12, 3.57], *P* = 0.04), 25% (2.03 CR10 [CI: 0.25, 3.80], *P* = 0.03) and 30% (1.82 CR10 [CI: 0.161, 3.48], *P* = 0.03) of MIP and significantly higher ratings of work/effort at 25% (1.67 CR10 [CI: 0.07, 3.27], *P* = 0.04) and 30% (1.37 CR10 [CI: 0.001, 2.73], *P* = 0.049) of MIP compared to females. There was no interaction between sex and *P*_mo_%MIP for dyspnoea unpleasantness (Figure 2b) or ratings of air hunger (Figure 2d) (both *P *> 0.05).

#### 3.2.2 Multidimensional dyspnoea profile

**FIGURE 3:**
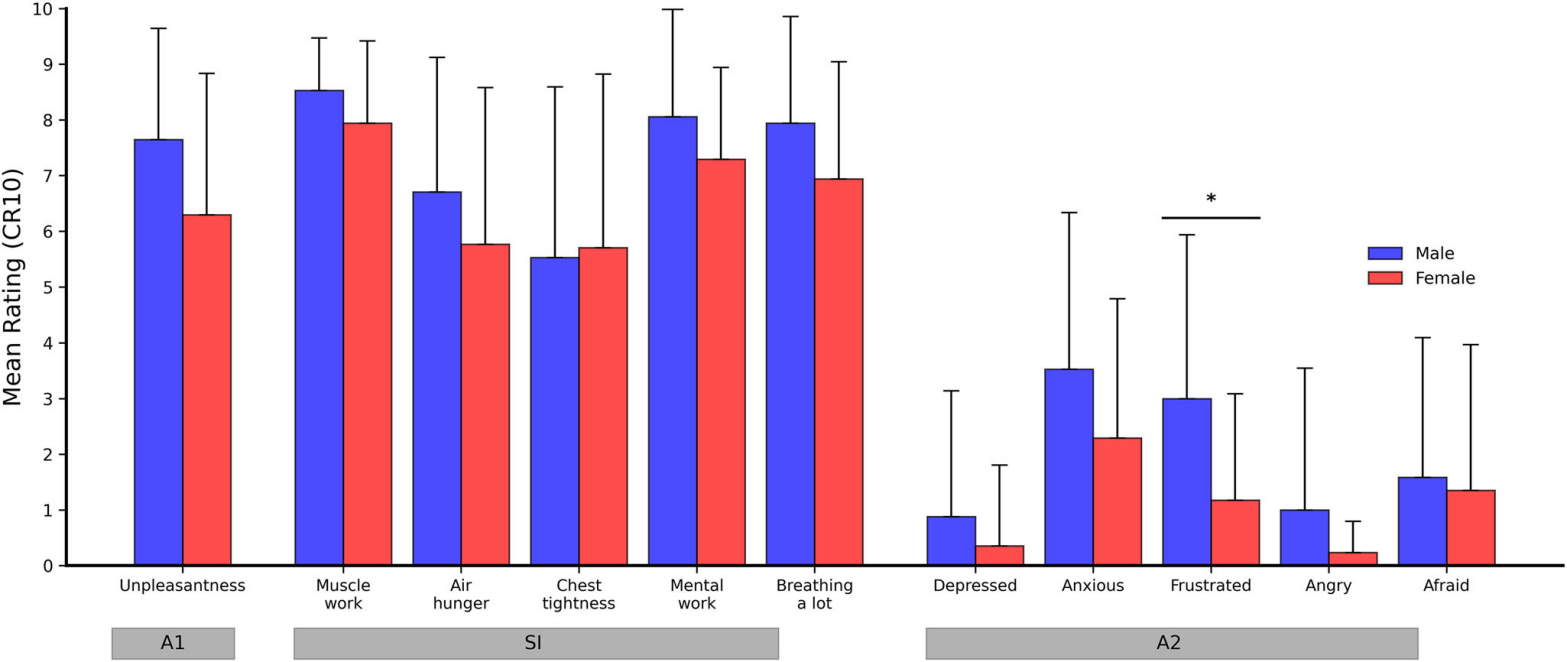
Multidimensional dyspnoea profile responses (CR10) at the end of IPTL. Values are means ± SD. Negative error bars are not shown to improve figure clarity. *P < 0.05: significant difference between sexes. A1, immediate unpleasantness; A2, emotional response; CR10, 0–10 category ratio scale; SI, sensory intensity.

Although males rated unpleasantness higher than females on average (M: 7.64 ± 2.00 CR10; F: 6.29 ± 2.54 CR10), this difference was not statistically significant (*P *> 0.05) (Figure 3). Males and females did not differ in their rating of the intensity of the sensory qualities of ‘muscular work’, ‘air hunger’, ‘chest tightness’, ‘mental effort’ or ‘breathing a lot’ (all *P *> 0.05) (Figure 3).

The intensity of feeling frustrated was rated significantly higher in males compared to females (M: 3.00 ± 2.94; F: 1.25 ± 1.95; *P* = 0.04). However, the intensity of feeling depressed, anxious, angry and afraid did not differ between the sexes (all *P *> 0.05) (Figure 3).

Males and females did not differ in the selection frequencies of the sensory qualities of ‘muscular work’, ‘air hunger’, ‘chest tightness’, ‘mental effort’ or ‘breathing a lot’ (all *P *> 0.05). The selection frequency of the most accurate SQ did not differ between sexes (*P *> 0.05).

#### 3.2.3 Reason for ending the trial

All males and all but two females stopped due to the inability to generate sufficient inspiratory pressure on three consecutive breaths despite verbal encouragement. The two females stopped due to intolerable breathing discomfort.

### 3.3 Physiological responses to IPTL

**FIGURE 4:**
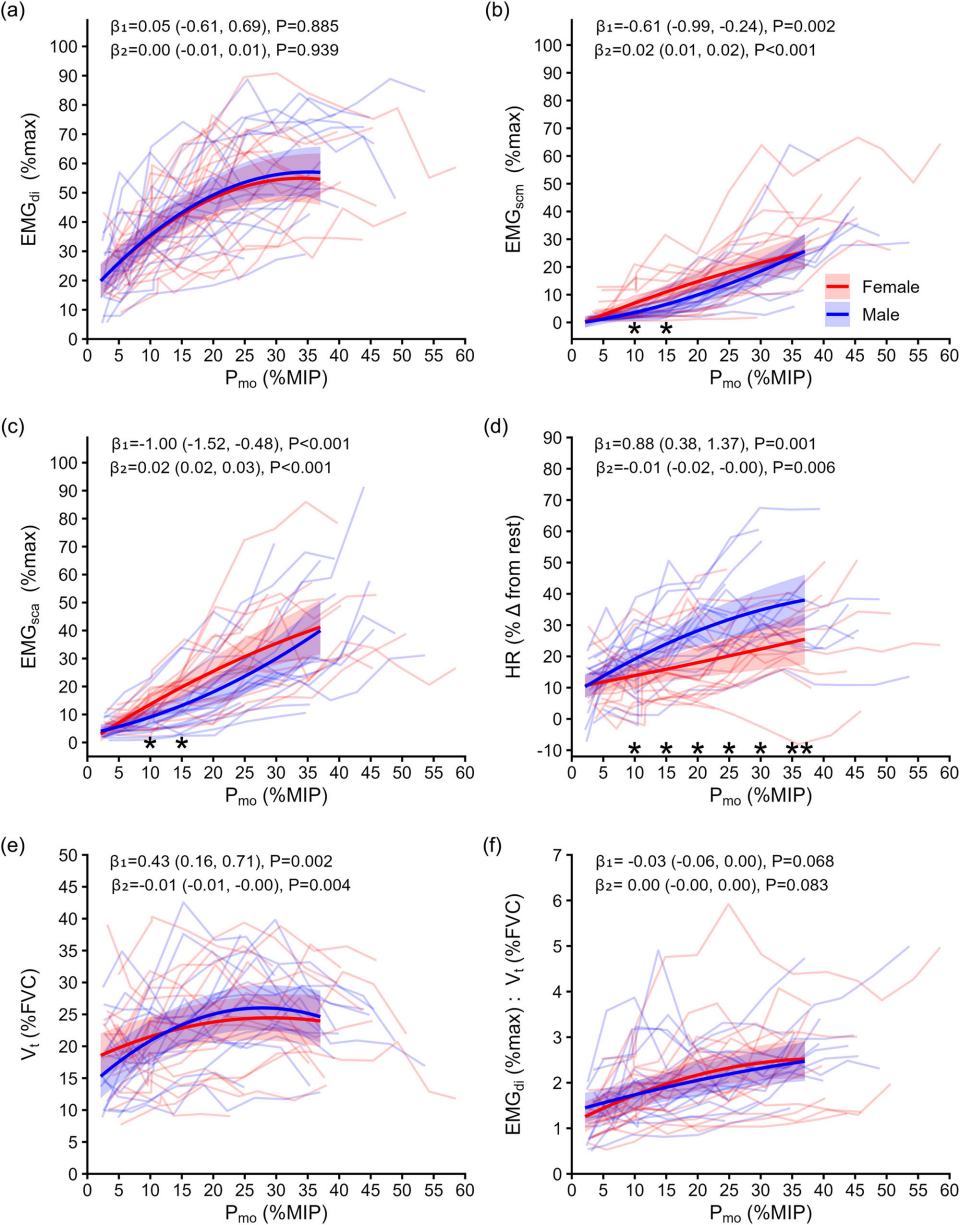
Physiological responses during IPTL in males and females. (a) Diaphragm activation as %maximum. (b) Sternocleidomastoid activation as a percentage of maximum. (c) Scalene activation as a percentage of maximum. (d) Percentage change in heart rate from rest. (e) Tidal volume as a percentage of forced vital capacity. (f) The ratio between diaphragm activation as a percentage of maximum and tidal volume as a percentage of forced vital capacity. Bold lines and shaded areas (95% CIs) represent model estimates and thin lines represent individual participant responses at each increment of *P*_mo_%MIP. Trendlines and 95% CIs from the models are truncated at the group mean peak *P*_mo_%MIP (group: 37.3 ± 9.0%; M: 37.2 ± 8.6%; F: 37.3 ± 9.7%). β_1_, linear interaction term (and 95% CIs) between sex and stimulus level; β_2_, quadratic interaction between sex and stimulus level. *P < 0.05: statistically significant between sexes estimated marginal mean contrasts. EMG_di_, diaphragm electromyography; EMG_sca_, scalene electromyography; EMG_scm_, sternocleidomastoid electromyography; FVC, forced vital capacity; HR, heart rate; *V*_t_, tidal volume.

There was no significant interaction for EMG_di_%max between sexes for the linear or quadratic effects in response to relative increases in *P*_mo_ (both *P *> 0.05) (Figure 4a). There was a significant interaction between sex and increasing *P*_mo_%MIP for both EMG_scm_%max (Figure 4b) and EMG_sca_%max (Figure 4c). The relationships for both muscles were characterized by a reduced initial linear slope (SCM: *P* = 0.002; SCA: *P *< 0.001) but a more pronounced quadratic increase in relative activation (SCM: *P *< 0.001; SCA: *P *< 0.001) in males compared to females. Accordingly, estimated marginal mean contrasts showed that, compared to females, males had lower activation of the SCM and SCA muscles when *P*_mo_ was 10% (SCM: −3.40% [CI: −6.34, −0.46], *P* = 0.025; SCA: (−4.57% [CI: −8.55, −0.58], *P* = 0.03) and 15% (SCM: −4.48 [CI: −8.32, −0.63], *P* = 0.024; SCA: −6.58% [CI: −12.2, −1.00], *P* = 0.02) of MIP.

There was a significant interaction between sex and both the linear (*P* = 0.001) and quadratic (*P* = 0.006) effects of increasing *P*_mo_%MIP on heart rate. This was characterized by a steeper initial rise but a more prominent plateau at higher *P*_mo_%MIP in males compared to females. Pairwise estimated marginal mean contrasts showed males had a higher heart rate as a percentage change from rest at 10% (5.29%Δ [CI: 0.20, 10.4], *P* = 0.04), 15% (8.06%Δ [CI: 2.17, 13.9], *P* = 0.009), 20% (10.2%Δ [CI: 3.17, 17.2], *P* = 0.006), 25% (11.6%Δ [CI: 3.37, 19.9], *P* = 0.007), 30% (12.5%Δ [CI: 2.80, 22.1], *P* = 0.01), 35% (12.6%Δ [CI: 1.44, 23.8], *P* = 0.03), and at the group mean peak *P*_mo_%MIP (12.5%Δ [CI: 0.537, 24.5], *P* = 0.04) compared to females (Figure 4d).

There was a significant interaction between sex and both the linear (*P* = 0.002) and quadratic (*P* = 0.004) effects of increasing *P*_mo_%MIP on *V*_t_%FVC, with males exhibiting a steeper initial increase and sharper decline in *V*_t_%FVC at higher *P*_mo_%MIP compared to females. There were, however, no significant differences in *V*_t_%FVC between sexes at any *P*_mo_%MIP (all *P *> 0.05) (Figure 4e). There was no significant linear or quadratic interaction between sex and *P*_mo_%MIP for NMD (both *P *> 0.05) (Figure 4f).

### 3.4 Perceptual responses to HERB

#### 3.4.1 CR10 ratings

**FIGURE 5:**
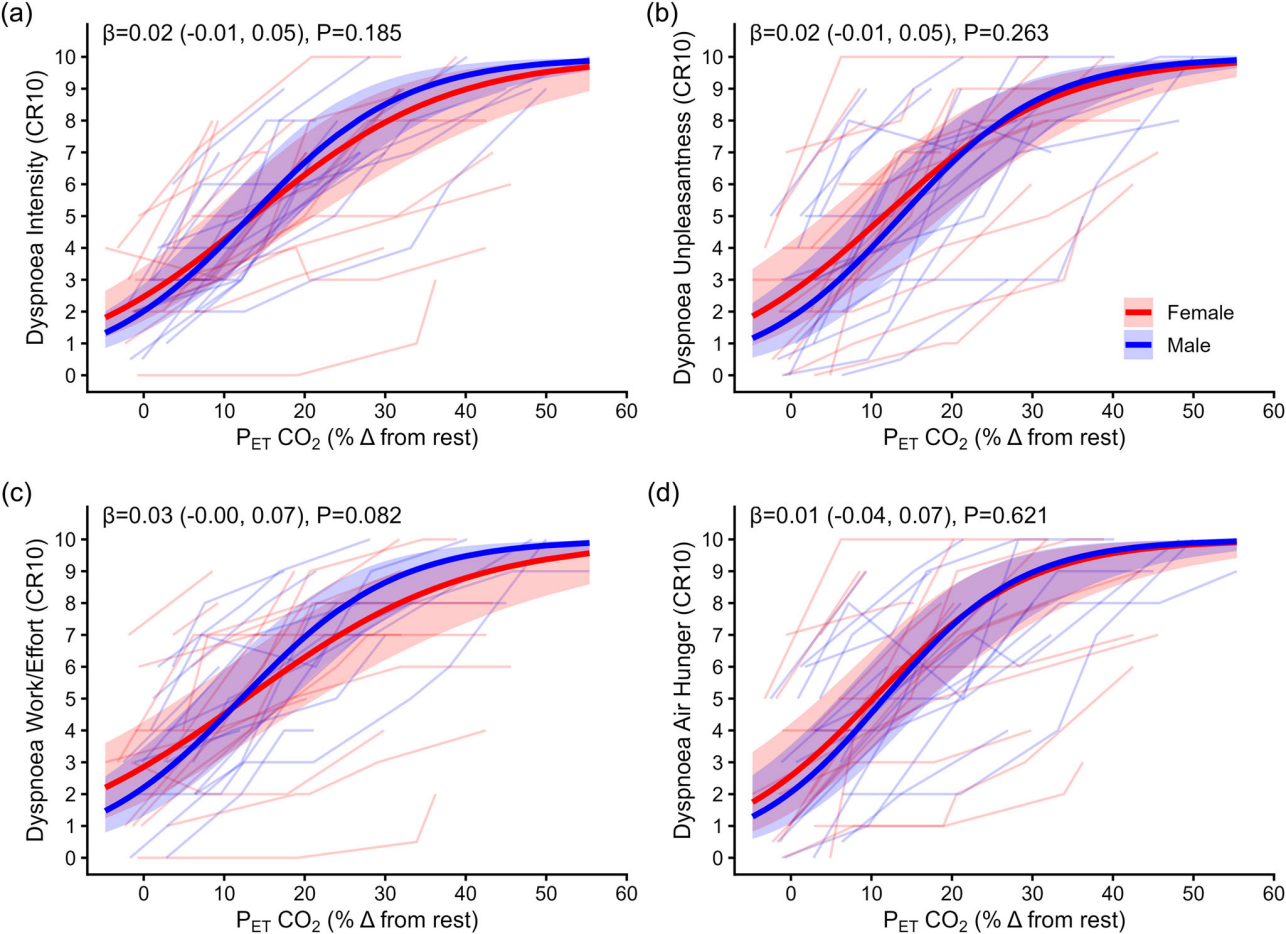
Dyspnoea responses during HERB in males and females. (a) Rating of dyspnoea intensity. (b) Rating of dyspnoea unpleasantness. (c) Intensity of the descriptor work/effort. (d) Intensity of the descriptor air hunger. Bold lines and shaded areas (95% CIs) represent model estimates and thin lines represent individual participant responses at each increment of %Δ$P_{\mathrm{ETC}O_{2}}$. β, change in log‐odds for males, compared to females per unit change in %Δ$P_{\mathrm{ETC}O_{2}}$.  CR10, 0–10 category ratio scale; HERB, hypercapnic expiratory resistive breathing; $P_{\mathrm{ETC}O_{2}}$, partial pressure of end tidal carbon dioxide.

Modelling of the perceptual responses to relative increases across the HERB trial is shown in Figure 5. There were no significant interactions between sex and %Δ$P_{\mathrm{ETC}O_{2}}$ for intensity, unpleasantness, work/effort of breathing or the sensation of air hunger (all *P *> 0.05).

#### 3.4.2 Multidimensional dyspnea profile

**FIGURE 6:**
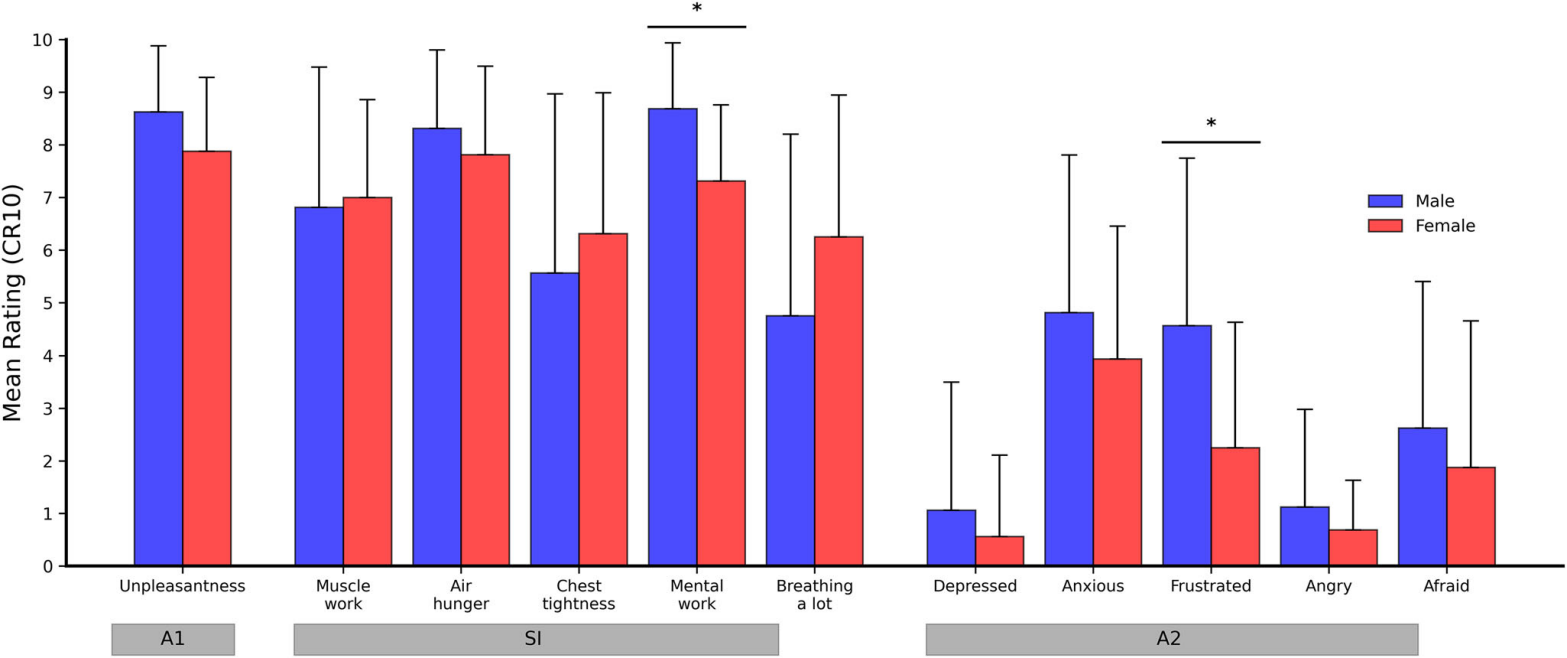
Multidimensional dyspnoea profile responses (CR10) at the end of HERB. Values are means ± SD. Negative error bars are not shown to improve figure clarity. *P < 0.05: statistically significant difference between sexes. A1, immediate unpleasantness; A2, Emotional response; CR10, 0–10 category ratio scale; SI, sensory intensity.

The mean rating of unpleasantness did not differ between males and females (*P *> 0.05) (Figure 6). Compared to females, males reported greater intensity of the descriptor ‘mental effort’ (M: 8.69 ± 1.25 CR10, F: 7.31 ± 1.45, *P* = 0.007) at the end of the HERB trial. Males and females did not differ in their rating of the descriptors ‘muscular work’, ‘air hunger’, ‘chest tightness’ or ‘breathing a lot’ (all *P *> 0.05).

The intensity of feeling frustrated was rated significantly higher in males compared to females at the end of the HERB trial (M: 4.56 ± 3.18 CR10, F: 2.25 ± 2.38 CR10, *P* = 0.03). However, the intensity of feeling depressed, anxious, angry and afraid did not differ between the sexes (all *P *> 0.05).

Males and females did not differ in the selection frequencies of the sensory qualities of ‘muscular work’, ‘air hunger’, ‘chest tightness’, ‘mental effort’ or ‘breathing a lot’ (all *P *> 0.05). Furthermore, the selection frequency of the most accurate SQ did not differ between sexes (*P* = 0.50).

#### 3.4.3 Reasons for ending the trial

The same proportion of males and females stopped the HERB trial due to intolerable breathing discomfort (*n* = 22), the remainder stopped due to an inability to keep *V*_t_ below the predetermined level for three consecutive breaths (*n* = 10). No participants reached a $P_{\mathrm{ETC}O_{2}}$ of 60 mmHg.

### 3.5 Physiological responses to HERB

**FIGURE 7:**
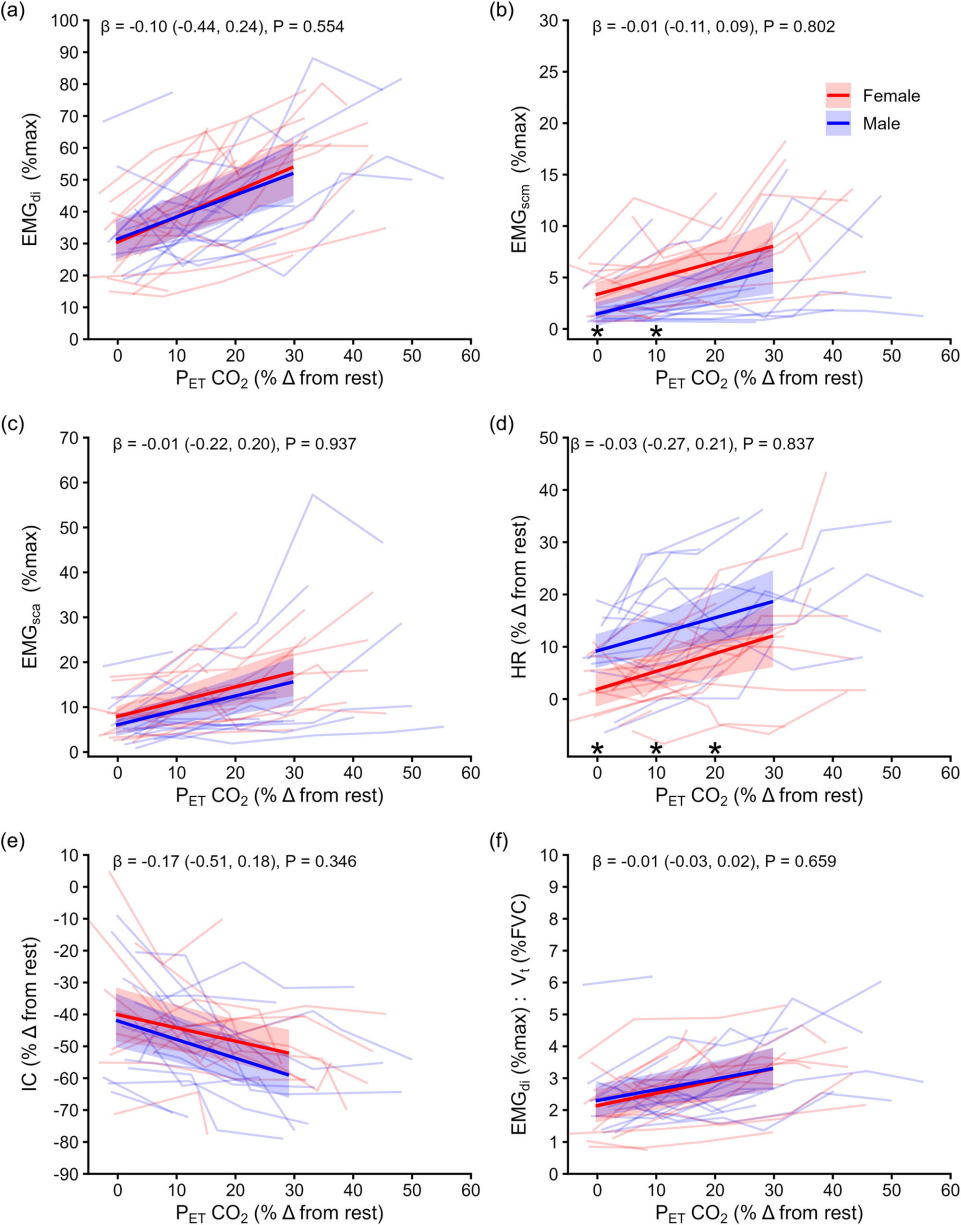
Physiological responses to HERB in males and females. (a) Diaphragm activation as a percentage of maximum. (b) Sternocleidomastoid activation as a percentage of maximum. (c) Scalene activation as a percentage of maximum. (d) Percentage change in heart rate from rest. (e) Percentage change in inspiratory capacity from rest. (f) The ratio between diaphragm activation as a percentage of maximum and tidal volume as a percentage of forced vital capacity. Bold lines and shaded areas (95% CIs) represent model estimates and thin lines represent individual participant responses at each increment of %Δ$P_{\mathrm{ETC}O_{2}}$. Trendlines and 95% CIs from the models are truncated at the group mean peak %Δ$P_{\mathrm{ETC}O_{2}}$ (group: 29.9 ± 12.9%; M: 30.5 ± 13.7%; F: 29.1 ± 12.2%). β, interaction term of each model. *P < 0.05 statistically significant between sexes estimated marginal mean contrasts. EMG_di_, diaphragm electromyography; EMG_sca_, scalene electromyography; EMG_scm_, sternocleidomastoid electromyography; FVC, forced vital capacity; HERB, hypercapnic expiratory resistive breathing; HR, heart rate; IC, inspiratory capacity; $P_{\mathrm{ETC}O_{2}}$, partial pressure of end tidal carbon dioxide; *V*_t_, tidal volume.

There was no interaction between sex and %Δ$P_{\mathrm{ETC}O_{2}}$ for EMG_di_%max (Figure 7a), EMG_scm_%max (Figure 7b), or EMG_sca_%max (Figure 7c) (all *P *> 0.05). Furthermore, there was no significant estimated marginal mean contrasts between sexes for any level of %Δ$P_{\mathrm{ETC}O_{2}}$ for EMG_di_%max and EMG_sca_%max. However, estimated marginal mean contrasts for EMG_scm_%max were significantly lower in males compared to females at 0% (−1.90% [CI: −3.64, −0.168], *P* = 0.03) and 10% (−2.03% [CI: −3.99, −0.074], *P* = 0.04) Δ$P_{\mathrm{ETC}O_{2}}$ (Figure 7b).

There was no interaction between sex and %Δ$P_{\mathrm{ETC}O_{2}}$ for percentage change in heart rate (*P *> 0.05); however, estimated marginal mean contrasts between sexes were significantly higher in males at 0% (7.34%Δ [CI: 2.58, 12.1], *P* = 0.004), 10% (7.09%Δ [CI: 1.81, 12.4], *P* = 0.01) and 20% (6.83%Δ [CI: 0.04, 13.6], *P* = 0.045) Δ$P_{\mathrm{ETC}O_{2}}$ (Figure 7d).

There was no interaction between sex and %Δ$P_{\mathrm{ETC}O_{2}}$ for percentage change in inspiratory capacity (Figure 7e) or NMD (Figure 7f). Estimated marginal mean contrasts between sexes were not significantly different at any %Δ$P_{\mathrm{ETC}O_{2}}$ for percentage change in inspiratory capacity or NMD (all *P *> 0.05).

### 3.6 Order effects

Effects of condition order on dyspnoea perception were assessed via the Mann–Whitney *U*‐test between condition order for ratings of the four CR10 questions at the highest stimulus level achieved by all participants (25% MIP for IPTL and 10% Δ$P_{\mathrm{ETC}O_{2}}$ for HERB) (Table 2). Across both trials, ratings on all four CR10 questions were consistently lower when a condition was completed first relative to second. This pattern reached statistical significance in the IPTL trial but not the HERB trial.

**TABLE 2:** CR10 dyspnoea ratings at highest stimulus level achieved by all participants separated by condition order.

	IPTL first	HERB first	*P*
**CR10 ratings at 25% MIP during IPTL**			
Intensity	5.28 ± 1.8	7.63 ± 1.9	0.002^*^
Unpleasantness	4.33 ± 2.1	7.06 ± 2.3	0.003^*^
Work/effort	6.00 ± 1.7	7.94 ± 1.7	0.001^*^
Air hunger	4.72 ± 2.1	7.00 ± 2.4	0.008^*^
**CR10 ratings at 10%Δ** $P_{\mathrm{ETC}O_{2}}$ **during HERB**			
Intensity	4.69 ± 2.0	4.47 ± 1.9	0.76
Unpleasantness	5.50 ± 2.8	4.70 ± 2.6	0.39
Work/effort	5.31 ± 2.4	5.00 ± 2.0	0.73
Air hunger	5.66 ± 2.5	5.00 ± 2.6	0.47

All values are presented as means ± SD. *Significant difference between sexes. Abbreviations: CR10, 0–10 category ratio scale; HERB, hypercapnic expiratory resistive breathing; IPTL: inspiratory pressure threshold loading; MIP: maximal inspiratory pressure; $P_{\mathrm{ETC}O_{2}}$, partial pressure of end tidal carbon dioxide.

## 4 DISCUSSION

The present study investigated sex differences in the perception of dyspnoea in response to breathing challenges that targeted sensations of work/effort and air hunger with the stimulus magnitude normalized within individuals. The main findings of this study are twofold: (1) during IPTL, males had a steeper increase in their ratings of dyspnoea intensity and work/effort of breathing, despite similar levels of EMG_di_%max and lower extradiaphragmatic activation compared to females; and (2) During HERB, there were no sex differences in perceptions of dyspnoea intensity, unpleasantness, the sensation of work/effort or the sensation of air hunger with concomitant between‐sex similarities in the physiological response to the trial.

### 4.1 IPTL

While we observed similar ratings of dyspnoea unpleasantness and air hunger between males and females, males had a steeper slope in ratings of dyspnoea intensity and work/effort with higher ratings for both at submaximal threshold pressures. Although only ratings of ‘frustration’ reached statistical significance, males consistently rated all aspects of the MDP higher than females, except for chest tightness, which was similar between sexes. Contrary to our hypothesis, these findings suggest a stronger perceptual response in males compared to females, when sensations of work/effort are targeted through IPTL.

We observed similar levels of NMD between males and females, which aligns with the absence of sex differences in dyspnoea unpleasantness and air hunger, consistent with our current understanding of the mechanisms underlying these sensations (Banzett et al., 2015). However, our findings suggest that the established relationship between NRD and dyspnoea does not fully account for the sex differences we observed in intensity and work/effort ratings during IPTL. First, our index of NRD, EMG_di_%max, was comparable between males and females and therefore cannot explain these perceptual differences. Second, although females exhibited greater extradiaphragmatic muscle activation, consistent with previous work (Mitchell et al., 2018; Molgat‐Seon et al., 2018), males reported higher dyspnoea ratings despite their lower relative extradiaphragmatic activation. This pattern runs counter to the typical association between NRD and dyspnoea, in which greater activation is expected to produce higher ratings of intensity and work/effort (Mendonca et al., 2014). A similar dissociation was recently reported by Mitchell et al. (2025), who found median work/effort ratings were 2 CR10 higher in males at peak exercise despite significantly lower EMG_di_%max compared to females, indicating that this observation is not unique to our IPTL model. Together, these discrepancies point to alternative or additional mechanisms, such as sex‐related differences in diaphragm fatigue or in how dyspnoea is anchored to previous experience when absolute stimulus intensity differs between groups.

One potential explanation for our observation of lower ratings of dyspnoea intensity and work/effort in females is the greater resistance to diaphragm fatigue in females compared to males, demonstrated during aerobic exercise (Guenette et al., 2010) and inspiratory loading (Geary et al., 2019; Leahy et al., 2024; Welch et al., 2018). Since diaphragm fatigue has been shown to amplify perceptions of dyspnoea without concomitant increases in relative diaphragm activation (Boyle et al., 2020), it follows that the sex differences observed in this study could be related, at least in part, to sex differences in diaphragm fatigue. Although we did not directly assess diaphragm fatigue in this study, we estimated the likelihood of diaphragm fatigue occurring using the time–tension index of the diaphragm (TTI_di_), where diaphragm fatigue is a function of both *P*_di_%max and the proportion of the duty cycle spent in inspiration (*T*_i_/*T*_tot_) (Bellemare & Grassino, 1982; Sheel et al., 2001, 2002). With the standardized duty cycle of 1 s in:1 s out (*T*_i_/*T*_tot _= 0.5), we observed a mean peak *P*_di_%max of 47% in males and 43% in females, indicating a mean peak TTI_di_ of ∼0.21–0.23. Bellemare & Grassino (1982) suggest a TTI_di_ of ∼0.20 can be maintained for 35–45 min and a TTI_di_ < 0.15 can be held indefinitely. In the present study, the duration of IPTL ranged from 9 to 19 min, with TTI_di_ >0.2 only reached in the latter stages of the trial. Therefore, it seems unlikely that the steeper slope and higher submaximal ratings of dyspnoea intensity and work/effort reflect greater fatigue in males compared to females, although we cannot definitively rule this out.

Another potential explanation for the sex difference in dyspnoea intensity and work/effort ratings during IPTL relates to the absolute magnitude of the pressure stimulus. Since males had higher MIP, each relative incremental increase in threshold pressure produced a greater absolute change in *P*_mo_ compared to females. Although this approach standardized the load relative to each participant's maximal inspiratory strength, the task itself was novel for most participants. Expectations and experience are known to influence the perception of breathlessness (Marlow et al., 2019), and it is therefore possible that participants anchored their ratings to previous real‐world experiences rather than to their maximal inspiratory strength. If males interpreted the larger absolute increments in *P*_mo_ as disproportionately greater increases relative to their past experience, this could contribute to the steeper rise in dyspnoea intensity and work/effort ratings observed in males. However, since perceptual anchors and expectations were not directly measured in this study, this postulation should be interpreted cautiously.

The discordance between relative increments and expectation is exemplified by the fact that eight participants rated one or more of the four dyspnoea questions on the CR10 scale as 10/10, which was defined to them as the ‘worst [intensity, unpleasantness, work/effort, air hunger] you have ever experienced or could imagine experiencing’ yet still completed subsequent stages, rating each increasing stimulus as 10/10. Assuming their ratings were anchored by this definition, their subjective understanding or expectation of what a 10/10 feels like changed throughout the trial. This indicates that despite a clear and standardized definition of a 10/10 on the CR10 scale, between‐subject differences in the experience with dyspnoea and expectations of the trial may have influenced perceptions of the absolute increases in *P*_mo_. Future work should use a familiarization trial to better anchor the participants’ maximal breathing discomfort.

### 4.2 HERB

Contrary to our hypothesis of greater perceptions of dyspnoea unpleasantness and air hunger in females compared to males, we found no sex differences in any of the four sensory descriptors asked during the HERB trial and only higher ‘mental work’ and ‘frustration’ in males on the MDP following the trial. We did not see any sex differences in NMD, which is in accordance with our understanding of the neurophysiological mechanism of air hunger. However, our results are in opposition to previous studies that have demonstrated sex differences in the perceptions of unsatisfied inspiration (synonymous with air hunger) in healthy young adults following whole body exercise (Cory et al., 2015) and during maximal hypercapnic rebreathing (Hall et al., 2025). An important distinction between our methodology and that of the previous studies is that we constrained *V*_t_ slightly above resting levels, whereas the others did not control or constrain the ventilatory response beyond the constraints naturally imposed by the individual's respiratory system at high levels of ventilation. By not allowing the ventilatory response to be dictated reflexively, we excluded and modified two key mechanisms involved in dyspnoea processing. First, by imposing a relative *V*_t_ constraint, we did not allow for the dyspnoea relieving effects of *V*_t_ expansion that occurs while free breathing during exercise or rebreathing (Flume et al., 1996; Vovk & Binks, 2007). Second, despite the elevated chemical drive to breathe from increased $F_{\mathrm{IC}O_{2}}$, our imposed fixed *V*_t_ and metronomic breathing required a greater degree of voluntary control of breathing to initiate and terminate inspiration and expiration at a fixed rate. Voluntary control of breathing affects dyspnoea differently than central control of breathing and may decrease perceptions of air hunger compared to reflex controlled breathing (Demediuk et al., 1992; Lansing et al., 2000; Moosavi et al., 2000). These key differences in experimental design may partially explain the lack of sex differences observed in the present study during the HERB trial, compared to previous studies. An alternative strategy to involuntarily constrain *V*_t_ expansion is mechanical restriction through external chest wall strapping to decrease FVC (Mendonca et al., 2014; O'Donnell et al., 2000). However, this approach may cause considerable chest discomfort, particularly in female participants, limiting its feasibility in the present study.

### 4.3 Heart rate response

During both IPTL and HERB, males had a consistently higher heart rate compared to females, except at 5% MIP during IPTL and 30%Δ$P_{\mathrm{ETC}O_{2}}$ during HERB, where heart rate was similar between sexes. This is consistent with previous respiratory loading studies which attributed the sex difference in heart rate to an attenuated sympathetic response in females, compared to males (Geary et al., 2019; Leahy et al., 2024; Niérat et al., 2017). However, the effect of sympathetic activity on dyspnoea remains unclear. In a small subset of participants, Niérat et al. (2017) showed no correlations between measures of heart rate or heart rate variability and breathing effort or discomfort.

### 4.4 Limitations

There is a large amount of overlap in the neurophysiology of the sensation of work/effort and air hunger, making the perceptual and physiological responses difficult to distinguish. As evidenced by the high CR10 ratings for all four questions for both IPTL and HERB trials, it is clear that high levels of work/effort and air hunger were experienced in both trials. Despite this, the key differences between the trial designs still represented different ‘types’ of dyspnoea stimulus (i.e., the rapid, large inspiratory efforts with no chemical stimulus in IPTL vs. low ventilation with a hypercapnic stimulus in HERB) and reflect the ‘mixed’ nature of dyspnoea, more similarly to how it is experienced in the real world (Lansing et al., 2009). Therefore, despite the high ratings for work/effort and air hunger during both trials, we are confident the methods used to induce dyspnoea target different neurophysiological pathways.

Another limitation of the present study is the use of EMG_di_ as a surrogate for NRD, as the two are not equivalent (Hudson et al., 2025). Intraoesophageal catheter‐derived EMG_di_ reflects activation of the crural diaphragm only, omitting the drive to extradiaphragmatic inspiratory muscles (Niro et al., 2021) and the costal diaphragm, which is preferentially recruited at higher levels of respiratory drive (Nguyen et al., 2020). Furthermore, EMG_di_ may more closely track mechanical ventilatory output than the efferent signal from the respiratory centres, meaning that chemoreflex‐driven changes in NRD may not be fully reflected in EMG_di_. Nevertheless, given that the diaphragm is the primary respiratory muscle and that EMG_di_ remains the closest feasible estimate of NRD in spontaneously breathing humans, it represents the most valid surrogate measure of NRD currently available.

As these respiratory loadings tasks were novel stimuli for our participants, anchoring dyspnoea ratings using our definition of 10/10 as ‘the most you have experienced or could imagine experiencing’ may have been challenging and may have resulted in some discordance between the relative stimulus and each participant's prior experience and expectations. Although a familiarization session could theoretically have provided a clearer reference for dyspnoea anchoring, we intentionally used a single‐session design to reduce participant burden, improve standardization, minimize day‐to‐day variability, and preserve participants’ naïveté to the respiratory loading tasks. This avoided perceptual learning and expectation effects that might have recalibrated their internal dyspnoea anchor, thereby altering how identical stimuli were rated during the experimental session.

Lastly, laboratory models of dyspnoea in healthy young individuals do not perfectly replicate the dyspnoea experienced in other populations (i.e., clinical and/or aged) in the real world. While the generalizability of our results is uncertain, it lays the groundwork for exploring sex differences and mechanisms of dyspnoea in clinical and older populations through laboratory models. For example, ageing and disease amplify the relationship between respiratory response and dyspnoea (see Guenette & Jensen, 2013), and may introduce a heterogeneity that can affect the scaling of physiological (e.g., MIP) and perceptual (e.g., experience/expectation) measures when making comparisons between the sexes. As such, within the context of the participant population, consideration of these physiological changes and assessment of experience and expectations may help elucidate the underlying physiological mechanism of sex differences in dyspnoea in these populations.

### 4.5 Conclusion

Our findings suggest that in response to incremental inspiratory loads relative to an individual's MIP, males increase their ratings of intensity and work/effort more quickly than females, while ratings of ‘unpleasantness’ and ‘air hunger’ remain similar between the sexes. This sex difference, however, could not be explained by sex differences in NRD, as estimated by EMG_di_%max. Furthermore, our observation of higher relative extradiaphragmatic muscle activation in females was counterintuitive to our understanding of the relationship between NRD and dyspnoea intensity. We speculate that since dyspnoea ratings are anchored to prior experience, males may have interpreted the similar relative, but larger absolute, increments in *P*_mo_ as greater departures from expectation, resulting in a steeper increase in ratings of intensity and work/effort in males compared to females. Additionally, the sensory and affective perceptions of dyspnoea were similar between sexes when dyspnoea was induced via a relative chemical stimulus in the context of relative constraint to *V*_t_ expansion.

## AUTHOR CONTRIBUTIONS

Jack R. Dunsford, Jordan A. Guenette, Paolo B. Dominelli, Yannick Molgat‐Seon and Dennis Jensen contributed to the conception and design of the experiment. Jack R. Dunsford, Olga Radivojevic, Alanna S. Hind and Olivia N. Ferguson conducted data collection. Jack R. Dunsford analysed the data. All authors contributed to the interpretation of data and critical revision of the work. All authors have read and approved the final version of this manuscript and agree to be accountable for all aspects of the work in ensuring that questions related to the accuracy or integrity of any part of the work are appropriately investigated and resolved. All persons designated as authors qualify for authorship and all those who qualify for authorship are listed.

## CONFLICT OF INTEREST

None declared.

## GENERATIVE AI STATEMENT

Generative AI tools were not used in the preparation of this manuscript

## Data Availability

Data are available from the corresponding author upon reasonable request.
